# Reply to: Misinterpretation of solid sphere equivalent refractive index measurements and smallest detectable diameters of extracellular vesicles by flow cytometry

**DOI:** 10.1038/s41598-021-03113-1

**Published:** 2021-12-17

**Authors:** George C. Brittain, Marc-André Langlois, Sergei Gulnik

**Affiliations:** 1 Beckman Coulter Life Sciences, Life Science Research, Miami, FL USA; 2 University of Ottawa Flow Cytometry and Virometry Core Facility, Ottawa, Canada; 3 Department of Biochemistry, Microbiology and Immunology, Faculty of Medicine, University of Ottawa, Ottawa, Canada; 4 uOttawa Center for Infection, Immunity and Inflammation (CI3), Ottawa, Canada; 5 Present Address: Genotix Biotechnologies, Inc., Sunnyvale, CA USA

**Keywords:** Immunological techniques, Imaging the immune system, Immunology, Biological techniques, Biotechnology

**replying to**: Van der Pol, et al.; *Scientific Reports* 10.1038/s41598-021-03015-2 (2021).

## Introduction

We appreciate the critical review of our article^1^ by van der Pol et al., as it provides more detailed insight into the mechanics of their modeling^2^. Fundamentally, most of their arguments boil down to a contrast between our real-world empirical data vs. their theoretical modeling, based on a variety of assumptions. We will briefly comment on their main points.

## Van der Pol et al. model anisotropic EVs as isotropic core–shells

The core–shell modeling that van der Pol et al. used to estimate the detection limit for extracellular vesicles (EVs) on the CytoFLEX is based on assumptions that disregard the anisotropic nature of EVs. The input factors for their model are all isotropic (homogeneous), including constant refractive indices (RIs) for both the membrane and lumen^2^. In contrast, the EV membrane is highly anisotropic (inhomogeneous), with dielectric properties that vary with size and curvature, and they should instead be using an appropriate anisotropic core–shell model to accurately calibrate flow cytometers in such a manner. Scientists and mathematicians have worked on phospholipid-bilayer vesicles for over 50 years to determine how to properly account for the effects of membrane anisotropy on light scatter and polarization, and many models do indeed exist^3–11^. Given that anisotropic core–shell models predict higher light-scatter efficiency than equivalently sized isotropic solid spherical particles, while isotropic core–shell models predict the opposite: lower light-scatter efficiency, this is a fundamental issue that may lead to inaccurate results, particularly for smaller EVs whose anisotropy increases with decreasing size.

**Figure 1:**
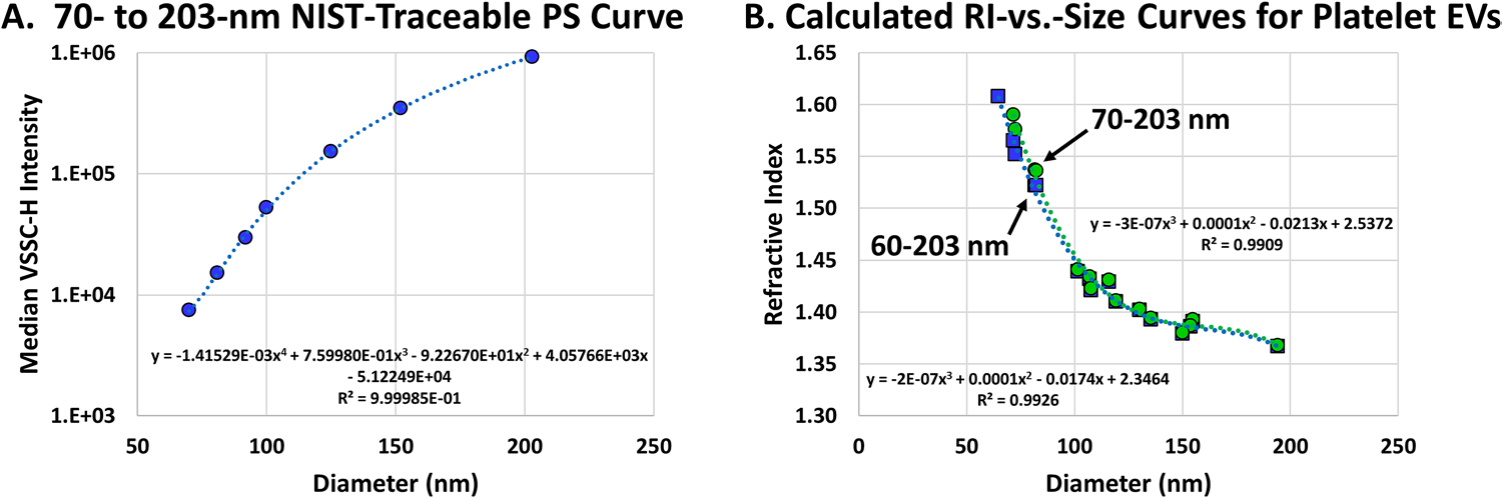
Comparing the RI-vs.-size curves calculated for plasma-derived platelet EVs using 60- to 203-nm vs. 70- to 203-nm NIST-traceable PS particles. (**A**) Fitting a median VSSC-H-vs.-diameter trendline to the 70- to 203-nm NIST-traceable PS-particle data from Supp. Table 2 in our article^1^. (**B**) An overlay comparing the RI-vs.-size curve calculated using 70- to 203-nm NIST-traceable PS particles (green) vs. the composite RI-vs.-size curve from Fig. 6C in our article^1^ (blue).

Liposomes, with a homogeneous membrane composition, have the least complexity of all phospholipid-bilayer vesicles—and thus the least anisotropy—as demonstrated by the lower RI-vs.-size curve in Fig. 1B of the van der Pol et al. letter^2^. The middle RI-vs.-size curve in Fig. 1B of their letter, representing EVs, was calculated using an isotropic core–shell model, combined with assumptions derived from literature values for homogeneous lipid films and measurements from the relatively linear 225- to 800-nm microparticle range—entirely outside of the exponential small-EV range being discussed^2^. Modeling the EVs as isotropic core–shells aside, both represent lower complexity and internal biophysical stresses than found in real-world small-EV membranes, and thus further bias the calculations toward reduced anisotropy. Notably, they did not discuss the various factors considered by anisotropic core–shell models, such as the differential radial vs. tangential dielectric permittivities^10,11^, the impact of the membrane anisotropy on the electrical field of the lumen^12^, etc. And they did not discuss the various nonlinear characteristics associated with EV membranes across the unique sub-200-nm size range, which has exponentially changing particle characteristics that may result in more complex dielectric phenomena, like the Maxwell–Wagner–Sillars effect^13–17^. Protein content is not the only contributing factor for increased light scatter. Moreover, proper validation of the extrapolated exponential region of their EV curve would require analyzing small-EV samples using an instrument that can detect in the sub-200-nm size range. The results from our own empirical analyses of small-EV samples using the CytoFLEX flow cytometer^1^ can be seen as the upper curves in Fig. 1B of the van der Pol et al. letter^2^. Ultimately, while their extrapolated EV curve does come closer to our empirical data than their liposome calculations, it does not address the anisotropic nature of the EV membrane, which we expect would shift the curve further upward.

Fundamentally, all Mie-theory models are complex approximations that need to be confirmed using empirical data, but oversimplification with the input assumptions may lead to inaccurate results for even the simplest mathematical models.

## Mie theory modeling for flow-cytometer standardization

Van der Pol et al. commented that our approach is unconventional^2^, however both approaches are based on similar Mie-theory calculations. They do not converge to the same results for the particles and viruses by chance.

In our article, the 60-nm polystyrene (PS) particles were resolved with a roughly tenfold dilution range where the population peak was distinguished from noise without swarming^1^. The minimal noise at the lower threshold was optical, resulting from light scatter off of H_2_O molecules and occasional impurities in the sheath solution. The peak APD electronic noise is located about a decade lower than the peak optical noise, with the upper tail about a decade lower than the threshold used. Importantly, any error associated with the physical detection and signal conversion for the 60-nm particles would certainly carry over to all of the other events, including the proximal nanoscale particles, and noise of any source impedes the resolution of small particles, which was not the case.

To clarify the matter, in Fig. 1 we eliminated the 60-nm PS particles from the standard curve and recalculated the EV RI-vs.-size curve from our article using the data for the 70- to 203-nm NIST-traceable PS particles found in Supp. Table 2 of our article^1^. Figure 1A shows the fitting of the trendline for the median VSSC-H-vs.-diameter data for the 70- to 203-nm PS particles, with the digits of the coefficients extended in scientific notation to improve the quantitative conversions. Figure 1B shows an overlay comparing the EV RI-vs.-size curve calculated using the 70- to 203-nm PS particles vs. the composite curve from Fig. 6C in our article^1^, which was calculated using 60- to 203-nm PS particles. Since the 64.8-nm EVs would require extrapolation outside of the fitted size range, they were not included in the recalculated curve. Ultimately, the two curves almost perfectly overlay one another, which demonstrates that our use of 60-nm PS particles in the standard curve to convert the EV light-scatter data does not explain the difference between our EV RI-vs.-size curve and the one calculated by van der Pol et al. using an isotropic core–shell model^2^.

## Fitting Gaussian curves to the flow cytometer data

While interesting, their approach of fitting Gaussian curves to the flow cytometer data is speculative, is based on their same modeling that they claim does not comport well with the nanoscale range, and the curves do not appear to fit the data in either shape or magnitude^2^.

We included a wide range of proximal VSSC-H intensities vs. RIs at equivalent sizes for each of the EV sample fractions in Supp. Figs. 11, 12, 13, and 14 in our article^1^, which demonstrate that even a median VSSC-H intensity difference as much as ± 23% would only result in a minimal difference in the calculated RIs of < 1.6%. To accommodate a RI of 1.45, in the middle of the range suggested by van der Pol et al., the Izon Fraction 8 can be calculated using our method to require median VSSC-H intensities 63.3%, 75.9%, 72.9%, or 82.8% lower for Donors 1–4, respectively, which would all be well below the threshold and would certainly prevent the detection of any of the samples. Yet, all of the sample peaks were reasonably well resolved.

In our article, to accommodate for the potential bias from the lower tail of the population distribution extending sub-threshold, the median VSSC-H intensities for each sample were derived from the central population distribution, gating to include the equivalent of approximately 1 standard deviation (SD) on either side of the center of the population distribution.

## Dynamic light scattering

Dynamic light scattering (DLS) is a well-established and standardized technique for particle-size analysis, and it has been used for decades. If the sample fraction is monomodal with a normal distribution, Cumulants fitting allows the hydrodynamic radius to be calculated using the Einstein–Stokes equation without making assumptions about the sample: the time-dependent fluctuations in the light scatter of particles due to Brownian motion depend only on the particle size and the temperature and viscosity of the sample buffer. The RIs of the particles need to be different from the buffer to distinguish the fluctuations, but they do not factor into the calculations unless the % Intensity measurements are converted to counts or volume distributions^18–20^, which we did not do.

**Figure 2:**
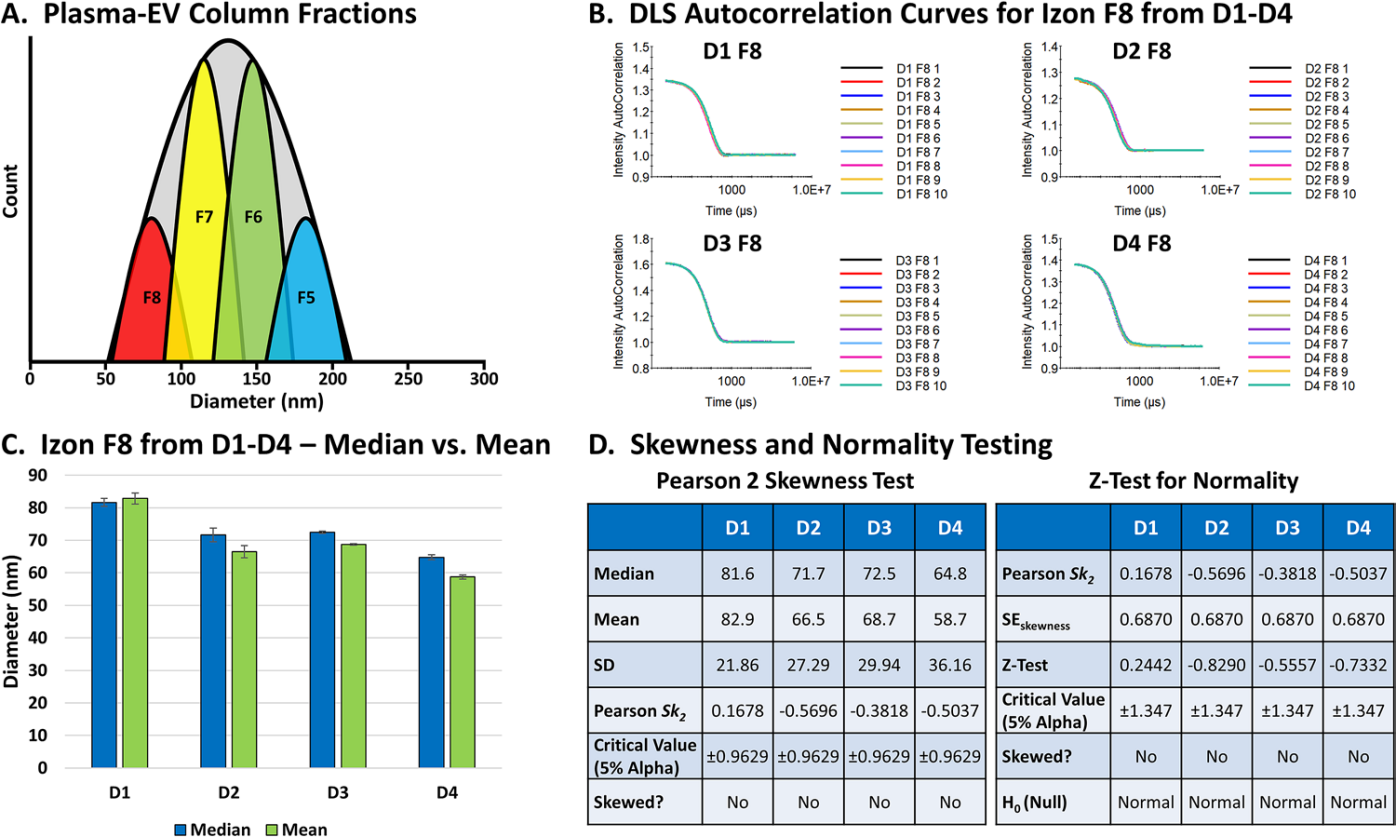
Analysis of the DLS measurements for the F8 EVs. (**A**) A depiction of the approximate EV size distributions using the fractionation approach in our article^1^. (**B**) The Cumulants autocorrelation curves for Izon F8 from D1–D4 by DLS. (**C**) The average median vs. mean diameters for Izon F8 from D1–D4 ± the SEM. (**D**) The Pearson 2 Skewness Test and *Z*-Test for Normality performed on the data for Izon F8 from D1–D4.

In our article, the sample inputs were physically fractionated to minimize the size distributions. The four fractions collected, F5–F8, were expected to span the narrow size range between approximately 70–200 nm based on the physical cutoffs (Fig. 2A), and the DLS data corresponded well with expectations^1^. These fractions contained EVs and certainly lipoproteins and other material as discussed in our article, but they were monomodal based on the physical size parameters of the fractionation approach.

In their letter, van der Pol et al. appear to emphasize “polydispersity” as a reference to multimodality, rather than the samples simply having a little wider size distribution than synthetic particle standards, which is the more relevant definition in this case. Reasonably monodisperse biological samples typically fall within the 0.1–0.5 polydispersity index (PDI) range, including manufactured liposomes^21–23^, and Cumulants analyses have been demonstrated to fit samples with PDI values up to 0.5 well without requiring higher-order cumulants (e.g., skewness and kurtosis)^24^. In our article, all of the samples had PDI values < 0.5, and the majority of the PDI values were at the lower end of the low/intermediate-polydispersity range (Supp. Fig. 10 in our article^1^). None of our samples were very broad or multimodal, much less discretely multimodal, as shown in the articles referenced by van der Pol et al.^25,26^.

Focusing in on F8 for Donors 1–4 (D1–D4), the autocorrelation curves for our DLS analyses were all smooth with a single exponential decay function within the appropriate signal-to-noise range, and with all error values < 0.005 (Fig. 2B). In addition, the sample medians and means were nearly overlapping (Fig. 2C), and the Pearson 2 Skewness Test and the *Z*-Test for Normality both support that statistically each sample had a normal distribution (Fig. 2D)^27,28^. The slight increases in PDI values for the fractions on the outer margins of the combined EV distribution, F5 and F8, were likely due to having slightly lower concentrations (Fig. 2A), but the measurements were all precise with low CVs^1^. Moreover, the determined sizes correlated well with the respective scatter intensities for each sample fraction, and, interestingly, the resulting VSSC-H-vs.-diameter data for each donor produced nearly perfect linear correlations (Fig. 6B in our article^1^). Our DLS analyzer was also calibrated using NIST-traceable silica particles and found to have near-perfect precision and accuracy (Supp. Fig. 15 in our article^1^). There is certainly some variability, as with any technique, but ultimately the combined methodology supports the accuracy on multiple levels.

## Comparison of the CytoFLEX flow cytometer to other technologies

Our article primarily focused on describing the optical-system design and characterizing the sensitivity limits of the different light-scatter detection modes on the CytoFLEX. Comparing the CytoFLEX to other technologies was outside of the scope of our article. While the Zhu et al. instrument is indeed very sensitive^29^, as has been demonstrated since the primary technology was first invented over 30 years ago^30,31^, it is not actually a flow cytometer, but rather a dedicated nanoparticle analyzer, optimized to function specifically within the nanoparticle range.

As a flow cytometer, the advantages of the CytoFLEX beyond its sensitivity are that:

1. Its event rate is roughly 1000 × faster than photon-counting approaches due to having a more rapid detector refresh rate, capable of analyzing nanoparticles at upwards of 1,000,000 events/min under optimal circumstances.
2. It has a dynamic light-scatter detection range for biological vesicles extending from roughly 60 nm to 100 μm, using a combination of 3 light-scatter detection modes^1^.
3. It is available with up to 6 lasers and 21 highly sensitive fluorescence channels (the EV analyses in our article used 5 fluorophores spread across 3 lasers^1^).
4. It is user-friendly, requiring minimal setup or maintenance.
5. And it can be fully automated with a plate-loader format.

The CytoFLEX can rapidly analyze both EVs and cells in the same sample with the full functionality of a flow cytometer, without any modifications or compromises made to enhance the nanoparticle detection sensitivity, which often comes at the expense of a reduced dynamic size range. While it would certainly be advantageous if the CytoFLEX could fully resolve the lower tail of the smallest EV populations, it is useful in many more contexts as simply a highly sensitive general-use flow cytometer.

## Conclusion

The main goal of our article was to describe the CytoFLEX design and detection modes, to characterize the light-scatter detection capabilities, to demonstrate some approaches for preparing and analyzing small particles, and to propose an alternative method for approximating particle size from light-scatter data. Ultimately, in view of the facts and arguments listed above, we believe that the assumptions and technical approaches that we used in our study were justified and valid. Furthermore, our results, calculations, and conclusions were all based on empirical data, without using any arbitrary values for the EV characteristics. This by no means excludes that there can be alternative and valid ways to carry out such experiments and data analyses, and to thereby also reach valid conclusions in accordance with an alternative set of clearly defined assumptions. However, in the case of EVs, we posit that such assumptions should consider the anisotropy of the phospholipid bilayer.
